# AGTR1 promotes lymph node metastasis in breast cancer by upregulating CXCR4/SDF-1α and inducing cell migration and invasion

**DOI:** 10.18632/aging.102032

**Published:** 2019-06-19

**Authors:** Yuxi Ma, Zihan Xia, Chunmei Ye, Chong Lu, Sheng Zhou, Juan Pan, Cuiwei Liu, Jieying Zhang, Tao Liu, Ting Hu, Linka Xie, Gang Wu, Yanxia Zhao

**Affiliations:** 1 Cancer Center, Union Hospital, Tongji Medical College, Huazhong University of Science and Technology, Wuhan 430022, China; 2 Department of Breast Surgery, Wuhan Women and Children’s Health Care Center, Wuhan 430022, China; 3 Department of Breast Surgery, Tongji Hospital, Tongji Medical College, Huazhong University of Science and Technology, Wuhan 430030, China; 4 Department of Pathology, Tongji Hospital, Tongji Medical College, Huazhong University of Science and Technology, Wuhan 430030, China; 5 Department of Pathology, Union Hospital, Tongji Medical College, Huazhong University of Science and Technology, Wuhan 430030, China

**Keywords:** breast cancer, lymph node metastasis, AGTR1, CXCR4, SDF-1α

## Abstract

The angiotensin II type I receptor (AGTR1) has a strong influence on tumor growth, angiogenesis, inflammation and immunity. However, the role of AGTR1 on lymph node metastasis (LNM) in breast cancer, which correlates with tumor progression and patient survival, has not been examined. AGTR1 was highly expressed in lymph node-positive tumor tissues, which was confirmed by the Oncomine database. Next, inhibition of AGTR1 reduced tumor growth and LNM in orthotopic xenografts by bioluminescence imaging (BLI). Losartan, an AGTR1-specific inhibitor, decreased the chemokine pair CXCR4/SDF-1α levels *in* *vivo* and inhibited AGTR1-induced cell migration and invasion *in* *vitro*. Finally, the molecular mechanism of AGTR1-induced cell migration and LNM was assessed by knocking down AGTR1 in normal cells or CXCR4 in AGTR1^high^ cells. AGTR1-silenced cells treated with losartan showed lower CXCR4 expression. AGTR1 overexpression caused the upregulation of FAK/RhoA signaling molecules, while knocking down CXCR4 in AGTR1^high^ cells downregulated these molecules. Collectively, AGTR1 promotes LNM by increasing the chemokine pair CXCR4/SDF-1α and tumor cell migration and invasion. The potential mechanism of AGTR1-mediated cell movement relies on activating the FAK/RhoA pathway. Our study indicated that inhibiting AGTR1 may be a potential therapeutic target for LNM in early-stage breast cancer.

## INTRODUCTION

Breast cancer is considered to have the characteristic of easily metastasizing to lymph nodes, which is a major prognostic factor in early-stage breast cancer [1, 2]. Patients with lymph node metastasis have a higher risk of loco-regional relapse and systemic metastases [3, 4]. The number of involved regional lymph nodes, along with tumor size and distant metastasis, forms the basis of the majority of tumor staging schemes [5, 6]. Axillary lymph node dissection (ALND) has been a classic adjuvant treatment for early-stage breast cancer patients with sentinel-node involvement. However, according to clinical trials IBCSG 23-01 [7] and ACOSOG Z0011 [8], ALND does not provide a survival benefit because of complications such as lymphoedema or limited arm movement. Therefore, radiotherapies, chemotherapies and targeted metastatic lymph node therapies may be prospective ways to control nodal disease and future distant dissemination.

Lymph node metastasis is a complex process that involves the migration of tumor cells towards tumor-associated lymphatic vessels, cell trafficking by chemokines and cytokines, successful seeding into draining lymph nodes and remodeling of the lymph node microenvironment. Chemokines [9], a superfamily of cytokine-like molecules, are secreted from organs and induce the directional migration of tumor cells that express specific chemokine receptors [10, 11]. A growing number of chemokine ligands and their receptors, including CCL19 and CCL21 for CCR7, CXCL13 for CXCR5, CCL1 for CCR8 [12, 13] and CXCL12 (also known as SDF-1α) for CXCR4, have been demonstrated to attract tumor cells to lymph nodes. Muller's study [14] suggested that CXCR4/SDF-1α particularly have prominent roles in primary and metastatic breast cancer, as well as a number of other important malignancies, including lung, brain, and prostate cancers. CXCR4 is highly expressed in human breast cancer cells, while SDF-1α exhibits peak levels of expression in organs such as the lymph node, lung and brain [15]. *In* *vivo*, neutralizing the interactions of CXCR4/SDF-1α significantly impairs breast cancer cell metastasis to regional lymph nodes [16–18].

A variety of biological molecules, including SIX1 [19], PDGF-D[18], and SEMA4C [20], have been implicated in lymph node metastasis. Apart from these factors, the angiotensin II (Ang II) type I receptor (AGTR1) may also affect lymph node metastasis. AGTR1, a component of the renin angiotensin system (RAS), which classically regulates cardiovascular homeostasis, has displayed the potential to stimulate cell growth [21–24], migration or invasion [25, 26] and to promote angiogenesis [27], inflammation and immunity [28, 29]. Accumulating evidence has revealed that the inhibition of AGTR1 by antagonists such as losartan [30, 31] and candesartan [32] can suppress angiogenesis, thereby contributing to the suppression of tumor growth and blood metastasis. However, the role of AGTR1 in lymph node metastasis of breast cancer has rarely been described.

There is a close correlation between Ang II/AGTR1 and CXCR4/SDF-1α, both of which are G protein-coupled receptor pathways, have the same downstream molecules and mediate cell migration [33]. In intrahepatic cholangiocarcinoma, activated hematopoietic stem cells (HSCs) promote tumor fibrogenesis, tumor progression and distant metastasis by mediating the epithelial-mesenchymal transition (EMT) via the Ang II/AGTR1 and CXCR4/SDF-1α axes [34]. Therefore, we hypothesized that the relationship between AGTR1 and CXCR4/SDF-1α has a great influence on lymph node metastasis in breast cancer.

To explore these unanswered questions, in the present study, we sought to identify the AGTR1 expression profile from clinical breast cancer samples. Then, we defined the oncogenic role of AGTR1 in lymph node metastasis *in* *vivo*. In addition, we examined the effect of the AGTR1 inhibitor losartan on CXCR4/SDF-1α expression in mouse models and tumor cells. Finally, we explored the potential mechanism of AGTR1-mediated cell migration and invasion and aimed to clarify whether losartan is beneficial for lymph node metastasis prevention and treatment in breast cancer.

## RESULTS

### The association of AGTR1 with clinicopathological data in breast cancer tissues

**Figure 1:**
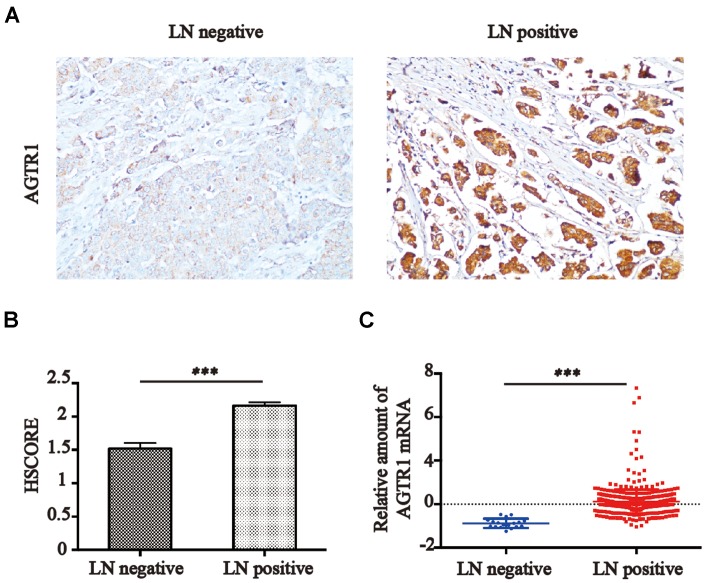
**AGTR1 expression is associated with lymph node metastasis.** (**A**) Representative images of AGTR1 expression in lymph node-negative or -positive tissues by IHC. (**B**) HSCORE of AGTR1 protein expression in breast cancer tissues from lymph node-positive or lymph node-negative patients. *** *P<*0.001. (**C**) Relative amount of AGTR1 mRNA in cancer tissues from lymph node-positive or -negative patients in the Oncomine database. *** *P<*0.001.

To investigate the relationship and significance between AGTR1 and clinical pathological factors, we conducted IHC staining in breast cancer tissue sections, and positive AGTR1 was observed in 275 cases. As indicated in Table 1, the number of lymph node-positive patients with AGTR1^high^ was 87, compared with 71 lymph node-negative patients with AGTR1^low^. Therefore, higher expression of AGTR1 protein significantly correlated with lymph node metastasis (*P*<0.05) but not with age, histological grade or tumor size. Representative images of IHC in lymph node positive and negative tissues are shown in Figure 1A. Quantification of AGTR1 by the HSCORE method (shown in Figure 1B) suggested a significant increase in its expression in breast cancer tissues with lymph node metastasis (2.16 in LN+ vs. 1.52 in LN-), while similar results were observed in ER+ or HER2- samples (Supplementary Figure 1).

**Table 1:** AGTR1 expression and clinicopathological data.

	**AGTR1**			
	**Number**	**High**	**Low**	***P* value**
**Age**				
≤50	174	106	68	0.3738
>50	101	56	45	
**Histological grade**				
I–II	239	144	95	0.2438
III	36	18	18	
**Tumor size (cm)**				
≤2	173	98	75	0.3208
>2	102	64	38	
**Lymph node status**				
negative	148	77	71	0.0123
positive	127	85	42	

We searched the Oncomine database for AGTR1 expression in breast cancer in lymph node-positive and lymph node-negative breast cancer patients and found that high expression of AGTR1 mRNA also occurred in lymph node-positive patients compared with lymph node-negative patients [0.12 vs. –0.88 (log2 median-centered intensity)] (Figure 1C). These results suggested that the upregulation of AGTR1 in primary tumors was associated with lymph node metastasis in breast cancer.

### Inhibition of AGTR1 reduces tumor growth and lymph node metastasis

**Figure 2:**
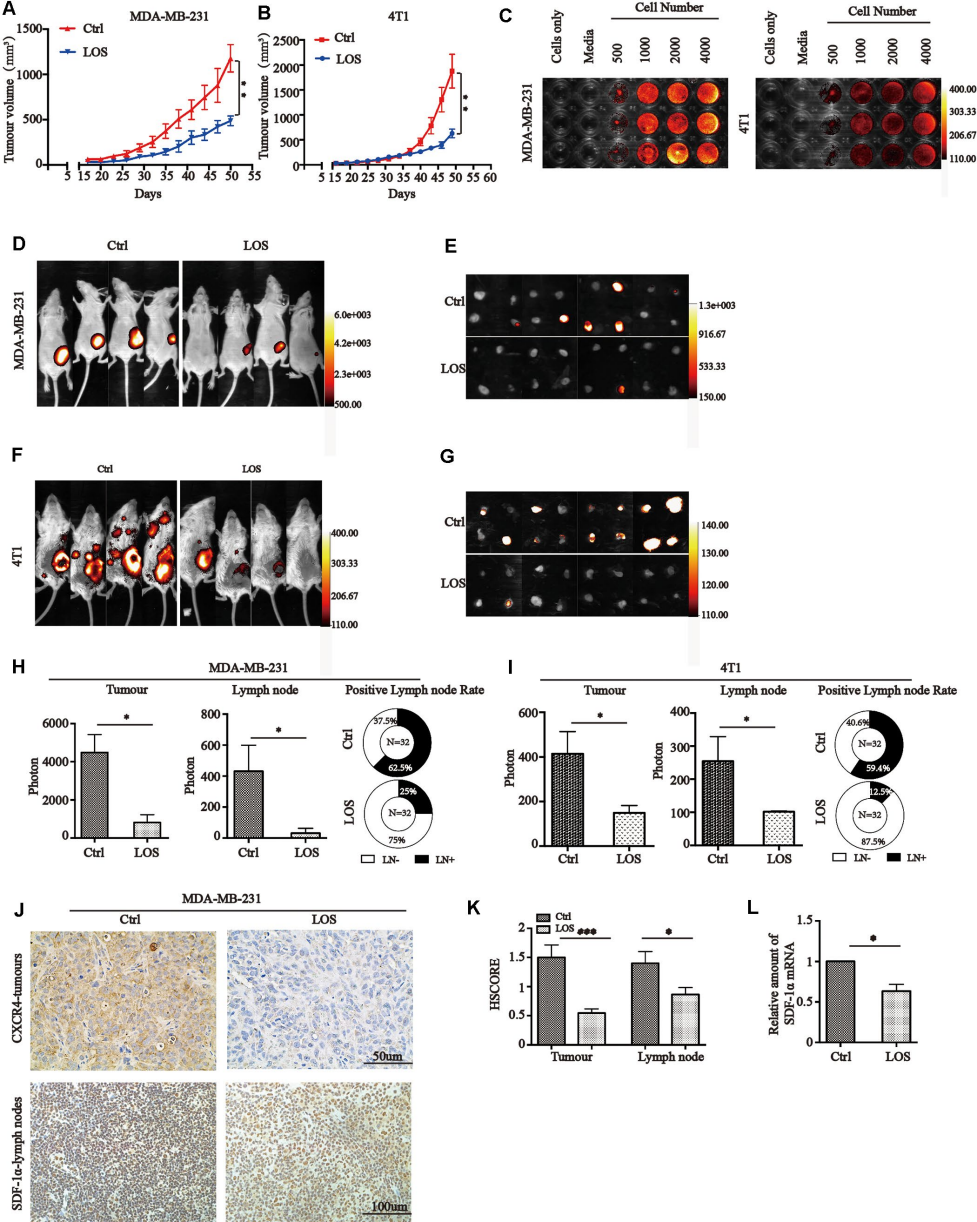
**Losartan reduces tumor growth and lymph node metastasis through CXCR4/SDF-1α *invivo*.** (**A**) and (**B**) MDA-MB-231 and 4T1 cells were injected into the fourth right mammary fat pad of nude mice and Balb/c mice. Two weeks after the injection, the tumor size was measured every 3 days. ** *P*<0.01. (**C**) BLI of MDA-MB-231 and 4T1 cells diluted in triplet wells from 4000 to 500 cells/well. Quantitative analysis of photon flux after adding luciferin substrate. (**D**) Representative images of MDA-MB-231 tumors and (**E**) lymph nodes for BLI analysis. Signal intensity was measured as photon flux (photons/second) and coded to a color scale. (**F**) Representative 4T1 tumor and (**G**) lymph node signal intensities were shown by BLI. The number of mice in each group was 8, and the total number of LN was 32. (**H**) and (**I**) Quantification of the signal intensities of tumors and lymph nodes and rates of lymph node metastasis in MDA-MB-231 and 4T1 tumors are shown below. * *P<*0.05. (**J**) Representative xenograft samples and lymph nodes images in different groups. Tissues were subjected to immunohistochemical staining with anti-CXCR4 or anti-SDF-1α. (**K**) HSCORE of CXCR4 and SDF-1α protein expression in mice tumor tissues and lymph nodes. * *P<*0.05, *** *P*<0.001. (**L**) Relative amount of SDF-1α mRNA in lymph node from different groups. * *P<*0.05.

To study the effect of AGTR1 on tumor growth and lymph node metastasis, we prepared two types of orthotopically implanted tumors, Balb/c-nu nude mice bearing MDA-MB-231 tumors and Balb/c mice bearing 4T1 tumors, and chose losartan, an AGTR1 antagonist, to block AGTR1. Saline or losartan (40 mg/kg, body weight) was administered by gavage (i.g.) every day in the control and losartan groups, respectively, for two weeks (mice with MDA-MB-231 tumors) or one week (mice with 4T1 tumors) after implantation. The growth curves showed that losartan administration resulted in a significant reduction in tumor growth (Figure 2A and 2B). In parallel, we used bioluminescence imaging (BLI) to monitor tumor growth in MDA-MB-231 and 4T1 tumors. Cells were transfected with lentivirus expressing the firefly luciferase gene and puromycin resistance gene. After selection with puromycin, the surviving colonies were screened under an *in* *vivo* imaging system (Figure 2C). We observed that orthotopically implanted tumors in the control group displayed significantly stronger firefly luciferase signals than those in the losartan group (Ctrl: 4482±947.6 vs. LOS: 819.8±404.1 in MDA-MB-231 tumors and Ctrl: 414.3±99.3 vs. LOS: 148.8±33.7 in 4T1 tumors) (Figure 2D and 2F).

To detect lymph node metastasis in mice bearing MDA-MB-231 and 4T1 tumors, we used indirect BLI to monitor four groups of lymph nodes (proper axillary, accessory axillary, subiliac, and popliteal lymph nodes) resected from mice. We observed that mice bearing MDA-MB-231 tumors in the control group developed metastases to lymph nodes with significantly stronger firefly luciferase signals (Ctrl: 430±169.8 vs. LOS: 31±31) (Figure 2E). Calculation of the number of positive lymph nodes from each group (total number of LN=32) showed that the proportion was 62.5% in the Ctrl group compared with 2.5% in the losartan group (Figure 2H). The same results were also observed in 4T1 tumors in which losartan treatment significantly decreased lymph node metastasis by quantitative analysis of the luciferase signal and the ratio of lymph node metastasis (Figure 2F and 2I).

### Inhibition of lymph node metastasis by AGTR1 antagonism through decreasing CXCR4/SDF-1α levels *in* *vivo*

Tumor cells preferentially disseminate to specific distant organs through chemokine guidance, which shares the same mechanisms as those regulating leukocyte homing and trafficking. The higher expression of CXCR4 on tumor cells and intensive generation of SDF-1α from target organs form a major pathway for breast cancer metastasis [35]. Therefore, in our study, we examined CXCR4 and SDF-1α expression in mice with orthotopically transplanted tumors using IHC and RT-PCR. Inhibition of AGTR1 decreased CXCR4 protein levels in tumors (1.50 in Ctrl vs. 0.55 in LOS) (Figure 2J and 2K) and SDF-1α levels in lymph nodes (Figure 2L). Taken together, these data suggest that blocking AGTR1 inhibits lymph node metastasis via the CXCR4/SDF-1α pathway.

### AGTR1 promotes tumor cell migration and invasion

**Figure 3:**
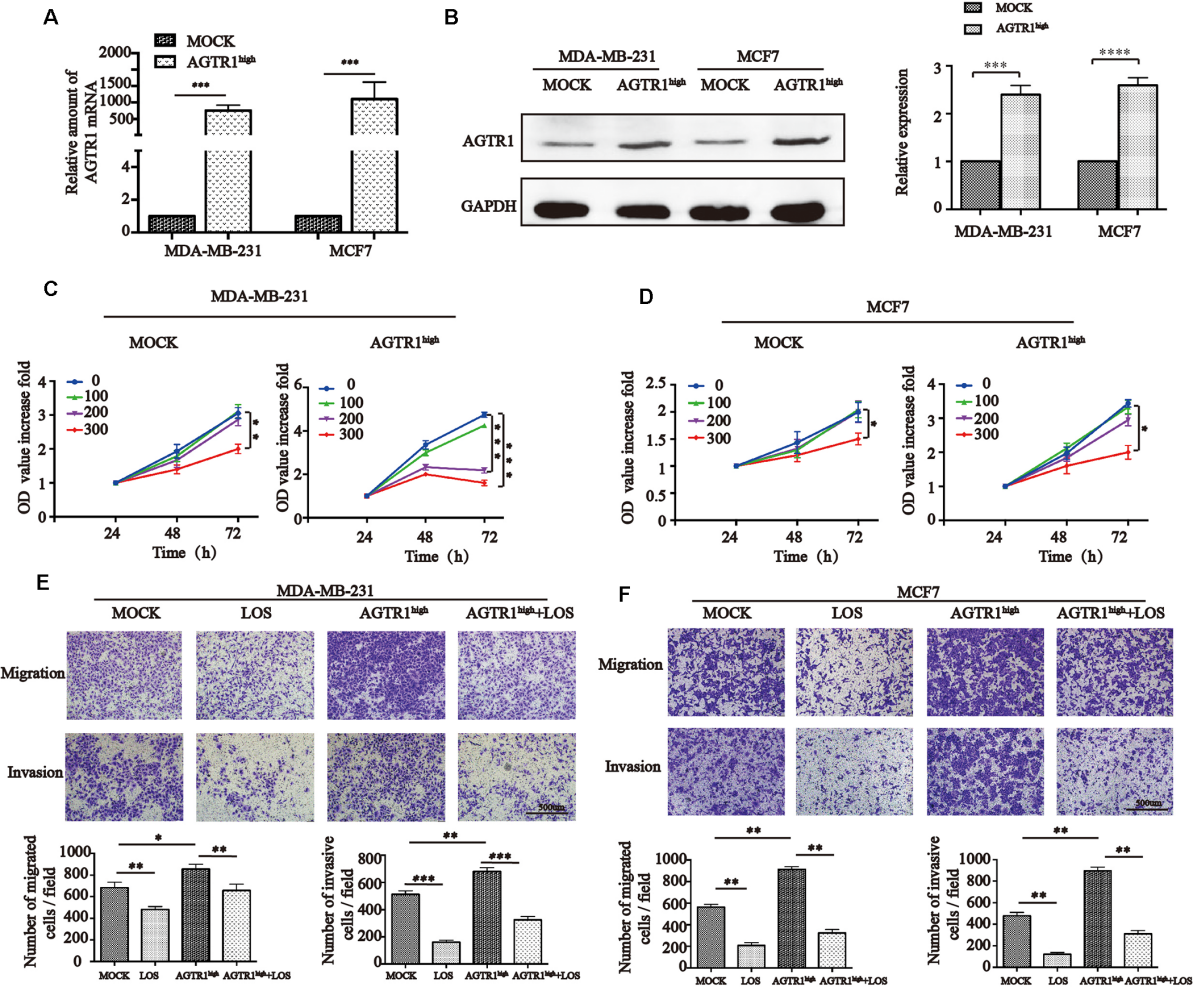
**AGTR1 promotes tumor cell proliferation, migration and invasion**. (**A**) RT-PCR and (**B**) Western blot analysis of AGTR1 overexpression after transfection into MDA-MB-231 and MCF7 cells. One representative Western blot image is shown, and the quantification of AGTR1 levels is provided in the right panels. *** P<0.001, **** P<0.0001. (**C**) and (**D**) MOCK and AGTR1^high^ cell viability after treatment with different concentrations of LOS (0, 100, 200, 300 μM) by CCK8. * *P<*0.05, ** *P*<0.01, *** *P*<0.001. (**E**) and (**F**) Effect of AGTR1 overexpression on cell migration and invasion. Images were captured with an inverted microscope (×100 magnification). The total number of migrating and invading cells in the fields was counted and is shown in the bottom panel. * *P*<0.05, ** *P<*0.01, *** *P<*0.001.

To investigate the role of AGTR1 in breast cancer cells, MDA-MB-231 and MCF7 cells were stably transfected with lentivirus expressing the AGTR1 gene (AGTR1 expression in breast cancer cells; see Supplementary Figure 2). Western blot and RT-PCR analyses were performed to confirm the stable overexpression of AGTR1 (Figure 3A and 3B). To evaluate the effect of losartan on cell viability, cells were treated with various concentrations of losartan (0, 100, 200, 300 μM) for 72 h. A significant killing effect on cell viability was observed at 300 μM of losartan in MOCK-MCF7 and MOCK-MDA-MB-231 cells (Figure 3C and 3D). We next examined the effect of AGTR1 overexpression on cell viability upon stimulation with losartan for 72 h. AGTR1-induced cell proliferation was suppressed by losartan challenge (200 μM) in AGTR1^high^ cells (Figure 3C and 3D). The results were consistent with the conclusions that AGTR1 promotes tumor growth in mouse models.

Cell migration and invasion are the essential mechanisms of lymph node metastasis in tumors, which may be the initial step in dissemination from primary tumors. To investigate the effect of AGTR1 overexpression on cell migration and invasion, transwell assays were performed. MDA-MB-231 (5×10^4^ cells/well) and MCF7 (2×10^5^ cells/well) cells were seeded into transwell upper chambers with 200 μl of serum-free medium and/or 100 μM losartan for migration and Matrigel invasive assays, while 600 μl of 20% FBS medium and/or 100 μM losartan was added to the lower chambers. After incubation for 16 h (MDA-MB-231) or 48 h (MCF7) at 37°C, as shown in Figure 3E and 3F, the number of AGTR1^high^-MDA-MB-231 and AGTR1^high^-MCF cells increased significantly on the bottom of the transwell membrane. In contrast, losartan treatment inhibited migratory and invasive abilities in both the MOCK and AGTR1^high^ groups. Similar to the results reported by Seo et al [36], AGTR1 accelerated the aggressiveness of breast cancer cells.

### AGTR1 promotes proliferation, migration, invasion and lymph node metastasis via CXCR4

**Figure 4:**
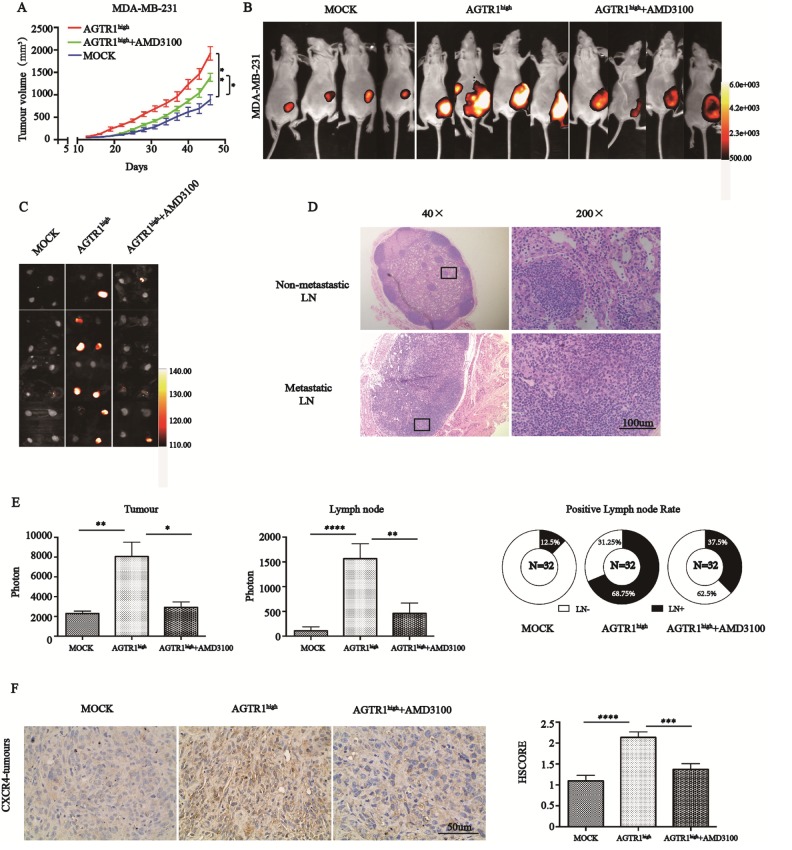
**AGTR1 contributes to lymph node metastasis via CXCR4.** (**A**) Growth curve of subcutaneous xenografts with MOCK and AGTR1^high^-MDA-MB-231 cells in nude mice. AMD3100 was administrated i.p. 14 days after implanting AGTR1^high^-MDA-MB-231 tumors. * *P*<0.05, ** *P<*0.01. (**B**) and (**C**) Representative images of xenograft models and lymph nodes are shown by BLI; n=8. (**D**) Representative HE staining figures of non-metastatic or metastatic lymph nodes in 4T1 mice. (**E**) Quantification of the signal intensities of tumors and lymph nodes and rates of lymph node metastasis in MDA-MB-231 tumors. * *P<*0.05, ** *P<*0.01, **** *P*<0.0001. (**F**) Representative xenograft tissues in three groups, which were subjected to immunohistochemical staining with anti-CXCR4. HSCORE of CXCR4 protein expression in mouse tumor tissues. * *P<*0.05, **** *P*<0.0001.

Based on the decrease of migration, invasion, lymph node metastasis and CXCR4 expression by losartan, it was hypothesized that AGTR1 plays its key role via CXCR4. We established an MDA-MB-231 xenograft model and found that the AGTR1^high^ group displayed a stronger capacity for growth (MOCK: 2299±241.5 vs. AGTR1: 8069±1451) and lymph node metastasis (MOCK: 108.5±80.18 vs. AGTR1: 1564±302.6) compared with MOCK-group tumors (Figure 4A–4C and 4E). AMD3100, a highly specific CXCR4 antagonist, is approved to suppress the growth and metastasis of many types of tumors. We administered AMD3100 to nude mice carrying AGTR1^high^-MDA-MB-231 tumors by intraperitoneal injection. The results revealed that AMD3100 could reverse AGTR1-induced tumor growth (AGTR1: 8069±1451 vs. AMD3100: 2923±557.1) and lymph node metastasis (AGTR1: 1564±302.6 vs. AMD3100: 458.9±208.4) significantly (Figure 4A–4C and 4E). IHC results showed that AMD3100 could also impede CXCR4 expression on AGTR1^high^ tumor tissues (1.37 in MOCK vs. 2.14 in AGTR1^high^ vs. 1.38 in AGTR1^high^+AMD3100) (Figure 4F). We conducted hematoxylin and eosin (HE) staining in each group of lymph nodes to verify tumor metastasis; representative images are shown in Figure 4D.

**Figure 5:**
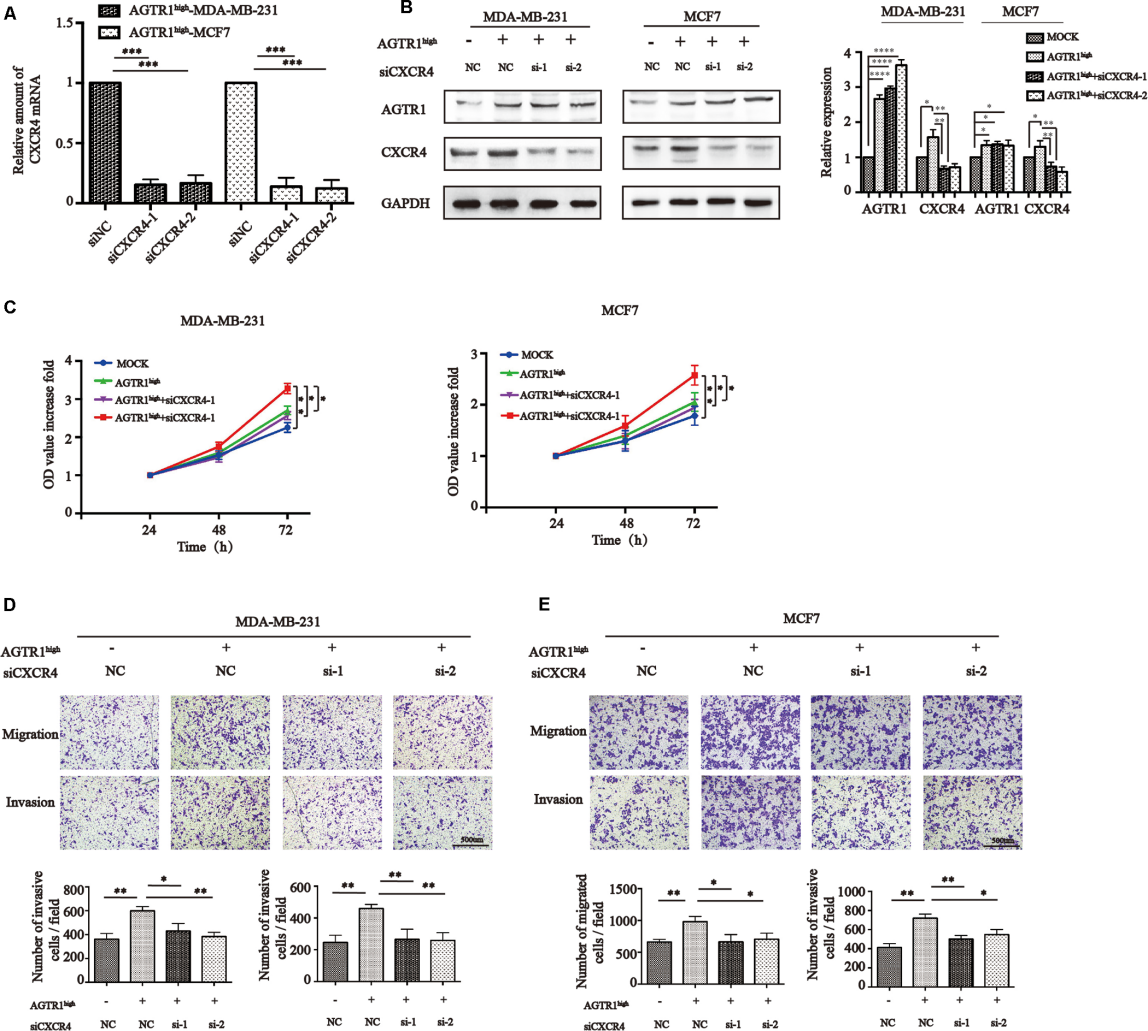
**AGTR1 increases proliferation, migration and invasion through CXCR4.** (**A**) RT-PCR and (**B**) Western blot analysis of CXCR4 knockdown in AGTR1^high^ cells. Representative pictures of Western blot of AGTR1 and CXCR4 expression and protein band intensities are shown. * *P<*0.05, ** *P<*0.01, *** *P*<0.001, **** *P*<0.0001. (**C**) Effect of AGTR1 overexpression and siCXCR4 on AGTR1^high^ cell proliferation using CCK8. * *P<*0.05, ** *P<*0.01. (**D**) Effect of AGTR1 overexpression and siCXCR4 on cell migration and invasion. The total numbers of migrating and invading cells in the fields were counted and are shown in the bottom panel. * *P*<0.05, ** *P<*0.01.

Further researches confirmed the results *in* *vitro* by knocking down CXCR4 in MDA-MB-231-AGTR1^high^ and MCF7-AGTR1^high^ cells using siRNA. Decreased CXCR4 expression was confirmed by RT-PCR and Western blotting (Figure 5A and 5B). CCK8 assays exhibited that AGTR1^high^ cells proliferated significantly faster than their MOCK cells in MDA-MB-231 and MCF7 cells, while siCXCR4 suppressed cells proliferation (Figure 5C). Transwell assays with MDA-MB-231 (2×10^4^ cells/well) and MCF7 (1×10^5^ cells/well) cells seeding into upper chambers revealed that the enhanced number of AGTR1^high^-MDA-MB-231 and AGTR1^high^-MCF cells on the bottom of the transwell membrane were inhibited significantly by the suppression of CXCR4 (Figure 5D and 5E). All together, AGTR1 accelerates proliferation, migration, invasion and lymph node metastasis through upregulating CXCR4.

### AGTR1 enhances CXCR4 expression *in* *vitro*

**Figure 6:**
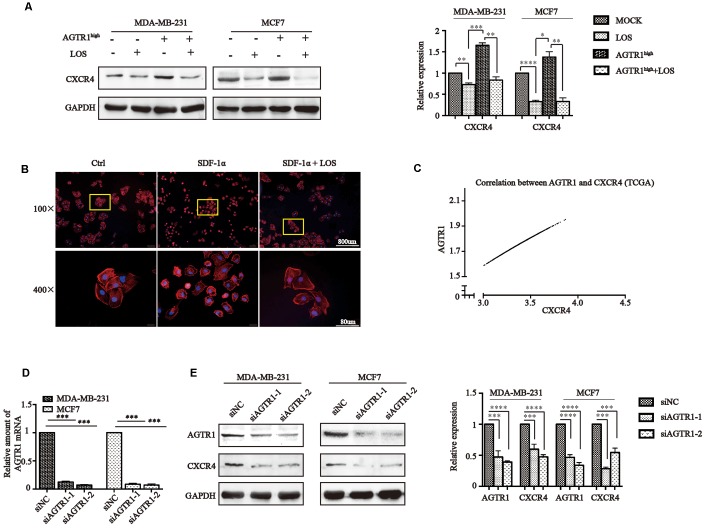
**AGTR1 induces the expression of FAK/RhoA signaling molecules through CXCR4.** (**A**) Effects of LOS and AGTR1 overexpression on CXCR4 expression in MDA-MB-231 and MCF7 cells detected by Western blot assay. Representative images are shown; protein bond intensities are in the right panel. * *P<*0.05, ** *P<*0.01, *** *P*<0.001, **** *P*<0.0001. (**B**) F-actin polymerization analysis in MDA-MB-231 stimulated with 100 nM SDF-1a or SDF-1a pretreated with LOS by rhodamine-phalloidin immunofluorescence. (**C**) Correlation between AGTR1 and CXCR4 analyzed by the TCGA database. (**D**) RT-PCR analysis of knocking down AGTR1 on MDA-MB-231 and MCF7 cells. *** *P*<0.001. (**E**) Representative figures of CXCR4 protein levels in siAGTR1 cells, as determined by Western blot assay. Quantification of AGTR1 and CXCR4 levels is in the right panel. *** *P*<0.001, **** *P*<0.0001.

From the results above, we found that losartan inhibited CXCR4 expression in mouse tumors. We next determined the effect of AGTR1 on CXCR4 expression *in* *vitro* by Western blotting. The results indicated that CXCR4 levels increased significantly in AGTR1^high^ MDA-MB-231 cells and MCF cells, which was inhibited by losartan (Figure 6A).

In tumor cells, high levels of actin polymerization are required for the formation of pseudopodia, which are necessary for the spreading of malignant cells into tissues and colonization. To verify the inhibitory effect of losartan on CXCR4, we treated MDA-MB-231 cells with SDF-1α and found that SDF-1α-treated tumor cells showed a distinct cytoskeletal redistribution of F-actin stress fibres and lamellipodia formation beginning 20 min after SDF-1α exposure by phalloidin staining. Pre-treatment of breast cancer cells with 100 μM losartan (24 h, 37°C) inhibited SDF-1α-induced actin polymerization and lamellipodia formation (Figure 6B). These results confirmed our findings that losartan blocked the effect of SDF-1α by inhibiting CXCR4.

To investigate whether the inhibition of AGTR1 expression can decrease CXCR4 expression, we knocked down AGTR1 in MDA-MB-231 and MCF7 cells using siRNA. Decreased AGTR1 expression was confirmed by RT-PCR and Western blotting (Figure 6D and 6E). The results showed that AGTR1 knock-down cells exhibited lower CXCR4 levels, indicating that the effect of siAGTR1 was the same as that of losartan (Figure 6A). Moreover, we searched the TCGA database and found that there was a significant correlation between AGTR1 and CXCR4 (R^2^=0.9814) (Figure 6C).

### AGTR1 induces the expression of focal adhesion kinase (FAK)/Ras homolog gene family member A (RhoA) signaling molecules

FAK is a cytoplasmic protein tyrosine kinase involved in cytoskeletal remodeling and cell adhesion structures [37, 38]. Together with FAK, RhoA and its downstream ROCKs are also involved in cell constriction and pseudopodia formation, which are required for cell migration [39, 40].

**Figure 7:**
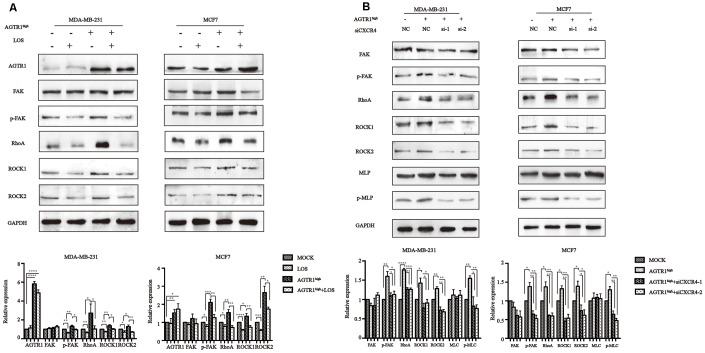
**The instrumental role of CXCR4 in AGTR1-mediated cell migration through the FAK/RhoA pathway.** (**A**) Effects of LOS and AGTR1 on the expression of FAK, p-FAK, RhoA, ROCK1, and ROCK2 analyzed through Western blotting. Representative images and protein bond intensities are provided in the bottom panel. * *P<*0.05, ** *P<*0.01, *** *P*<0.001, **** *P*<0.0001. (**B**) Effects of CXCR4 knockdown on the expression of FAK, p-FAK, RhoA, ROCK1, ROCK2, MLC and p-MLC in AGTR1^high^ cells, as determined by Western blotting. Representative images and protein bond intensities are provided in the bottom panel. * *P<*0.05, ** *P<*0.01, *** *P*<0.001, **** *P*<0.0001.

We therefore determined whether FAK/RhoA signaling pathways are activated in AGTR1-overexpressing tumor cells. Western blotting showed that p-FAK, RhoA, ROCK1 and ROCK2 levels increased significantly in AGTR1^high^ cells, while losartan decreased these levels in both MOCK cells and AGTR1^high^ cells (Figure 7A).

**Figure 8:**
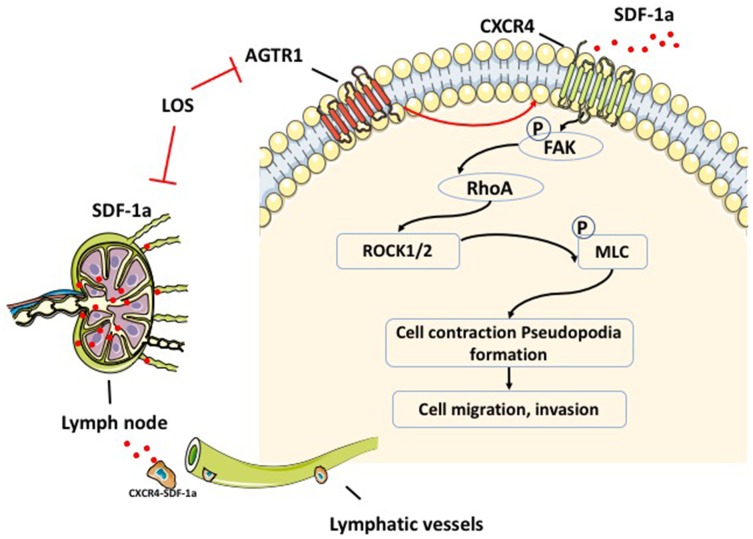
**Schematic description of AGTR1-mediated signaling through CXCR4/SDF-1α, which regulates breast cancer migration and lymph node metastasis.** AGTR1 enhances the level of SDF-1a in the lymph node, which attracts tumors that highly express CXCR4 cells. The mechanism of AGTR1-induced migration and invasion of tumor cells through upregulating CXCR4 and FAK/RhoA molecules.

### CXCR4 is crucial for AGTR1-induced FAK/RhoA molecule upregulation

For breast cancer, studies have provided evidence for the direct involvement of FAK and members of the Rho family in the CXCR4/SDF-1α-induced migration of tumor cells. The above results showed that AGTR1 regulated the expression of CXCR4 and FAK/RhoA signaling molecules. In this experiment, we attempted to clarify whether AGTR1 can activate the FAK/RhoA signaling pathway through CXCR4.

Cells with CXCR4 knock-down showed lower p-FAK, RhoA, ROCK1, ROCK2 and p-MLC levels, which were increased by overexpressing AGTR1 (Figure 7B). These results demonstrate that CXCR4 signaling is critical for AGTR1-induced lymph node metastasis.

## DISCUSSION

The RAS is frequently dysregulated in malignancy, which correlates with poor patient outcomes [41]. The main enzymatic components of the RAS include renin and angiotensin converting enzyme (ACE). The main product of ACE, Ang II, exerts a major effect via binding with specific AGTR1. Vinson first reported in early 1995 that AGTR1 had a possible link with breast cancer and later confirmed this finding in many tumor types, such as ovarian cancer, prostate cancer, and pancreatic cancer [42]. Losartan, an angiotensin-receptor blocker (ARB) that is widely used in the cardiovascular system, has great effects in preventing tumor progression in mice with induced breast cancer. However, there has been debate about the therapeutic role of ARBs in cancer patients over the years. One meta-analysis [43] suggested that ARBs were associated with a modestly increased risk of a new cancer diagnosis, while a more recent meta-analysis [44] confirmed that there was no difference in the risk of cancer with ARBs. Therefore, additional studies are needed to define the effect of ARBs on cancer incidence and mortality.

It has been extensively published that AGTR1 levels may correlate with tumor size (T stage) and distant metastasis (M stage) in some cases [45–48]. However, with regard to lymph node metastasis (N stage), Puddefoot [49] and Rodrigues [26, 50] revealed that Ang II reduces cell adhesion and metastasis, which correlates with lymph node metastasis indirectly. Here, we showed a direct function by which AGTR1 is involved in promoting lymph node metastasis. We found that AGTR1 expression in lymph node-positive patients was higher than that in lymph node-negative patients in our clinical samples and the Oncomine database. Moreover, the inhibition of AGTR1 decreased lymph node metastasis in orthotopic MDA-MB-231 and 4T1 breast carcinomas.

Lymphatic metastasis is regulated at multiple steps, including lymphangiogenesis, the upregulation of chemokines, and the migratory and invasive ability of tumor cells [9]. Tumor-associated lymphangiogenesis driven by VEGF-C promotes metastasis by providing routes for tumor cells to lymph nodes [51–55].

Interestingly, in our study, we did not find significant changes in VEGF-C mRNA in different mouse tumors (Supplementary Figure 3). A well-known hypothesis called ‘seed and soil’ describes the crosstalk between tumor cells and the microenvironment [56]. During this process, chemokines play a critical role in guiding tumor cells (the ‘seed’) to lymph nodes (the ‘soil’) [57]. Among all of these chemokines in breast cancer, CXCR4/SDF-1α is a major chemokine pair involved in lymph node metastasis [1]. CXCR4 is expressed by almost all cancer types, suggesting that the CXCR4/SDF-1α pair may be involved in site-specific metastasis formation in a large number of malignant diseases [58]. Therefore, we first investigated whether AGTR1 regulated lymph node metastasis through CXCR4/SDF-1α. *In vivo*, we found that losartan decreased CXCR4 levels in tumor tissues and SDF-1α at the mRNA level in lymph nodes. We found that blocking CXCR4 using AMD3100 on the basis of AGTR1-overexpression tumors could abolish AGTR1-induced lymph node metastasis, suggesting that CXCR4 signaling directly mediates AGTR1-induced metastasis.

Previous studies have shown that neutralizing the interactions of CXCR4/SDF-1α leads to a significant inhibition of lymph node and lung metastasis [1, 59]. Therefore, our data strongly suggest that blocking AGTR1 reduces lymph node metastasis *in* *vivo* and that this effect is likely mediated via CXCR4/SDF-1α. In addition, SDF-1α binds to CXCR7, another chemokine receptor that is highly expressed on breast cancer cells, and enhances CXCR7-mediated tumor migration and metastasis by activating STAT3, MMP9, MMP2 and VCAM-1 [60]. Apart from CXCR4/SDF-1α, CCR7- CCL19/CCL21 [61] are also key players in cell dissemination via the lymphatic system, but the level of CCL21 in lymph nodes was not influenced by losartan in our study (Supplementary Figure 4).

Another essential mechanism for inducing lymphatic metastasis is the migratory and invasive capacity of tumor cells [9]. Our observations revealed that AGTR1 accelerated breast cancer cell migration and invasion. There is evidence that in certain cancer types, such as gastric cancer, ovarian cancer, lung cancer and choriocarcinoma, Ang II/AGTR1 signaling is associated with the upregulation of a range of target genes that play a role in MMP-2 and MMP-9 activation and the induction of ICAM-dependent adhesion, inducing cell migration and EMT. EMT exhibits a disruptive effect on cell-cell junctions and promotes invasion into lymphatics, which was first revealed in studies of embryo implantation and embryogenesis [62, 63]. Our findings were consistent with those results.

Using orthotopically implanted mice, we found that losartan decreased CXCR4 expression. Therefore, *in* *vitro*, we validated those results and further clarified the relationship between AGTR1 and CXCR4 using RNA interference to knock down AGTR1. Both losartan and siAGTR1 decreased CXCR4, which indicated that AGTR1 may regulate the downstream molecule CXCR4. Rizzo's research has also revealed the close relationship between Ang II/AGTR1 and SDF-1α/CXCR4 in kidney diseases, indicating that increased Ang II could activate podocytes to release SDF-1α, which binds to its receptor CXCR4 located on parietal epithelial cells (PECs) [64]. A variety of CXCR4 inhibitors, including TI40, TNI4003, AMD3100, and AKBA, have also shown encouraging anticancer effects in pre-clinical studies [65]. In fact, CXCR4 antagonists have been translated into clinical trials, as presented at the 2017AACR-NCI-EORTC International Conference. X4P-001-IO, a CXCR4 inhibitor, in combination with axitinib has exhibited a promising disease control rate (DCR) of 93% in clear cell renal cell carcinoma, which was announced by Atkins' team. In HER2-negative metastatic breast cancer, the CXCR4 inhibitor balixafortide in combination with eribulin in phase 1 clinical trials exhibited a longer progression-free survival (PFS) than that for eribulin only; the one-year overall survival rate was 76% (balixafortide plus eribulin) vs. 54% (eribulin) [66]. From our study, we determined that losartan could inhibit CXCR4 expression in both mouse tumors and cells and may exhibit a similar therapeutic effect as CXCR4 inhibitors. Losartan may provide a safer and more economic strategy for CXCR4-induced lymph node metastasis and relapse.

SDF-1α mediates actomyosin cytoskeletal rearrangements and thereby regulates cell migration [67]. After pretreating MDA-MB-231 cells with losartan and then SDF-1α for 20 min, we found that losartan reduced SDF-1α-induced pseudopod formation by phalloidin staining. This effect validated that losartan decreased CXCR4 expression on cell membranes, which reduced the interaction between CXCR4 and SDF-1α. Actually, SDF-1α expression is higher in lung, pancreatic, and esophagogastric tumors and is associated with hypoxia-induced angiogenesis [68]. However, SDF-1α expression is lower in breast tumors, and these differences are reasonable because high levels of SDF-1α in primary tumors promote local invasion, which contributes to poor survival [69]. Low SDF-1α in breast cancer promotes its migration to distant organs. In our study, we showed that losartan could decrease both CXCR4 in primary tumors and SDF-1α in lymph nodes, which is an essential mechanism for reducing lymph node metastasis in breast cancer.

FAK/RhoA signaling molecules are widely accepted as important promoters of directional cell movement [38, 39]. Therefore, we investigated whether AGTR1 affects the FAK/RhoA signaling pathway. Our results showed that AGTR1 increased FAK/RhoA molecules and their downstream effectors ROCK1, ROCK2 and MLC, which agreed with previous studies showing that FAK/RhoA is activated by Ang II/AGTR1. FAK, which upon Ang II-induced activation translocates to sites of focal adhesion with the extracellular matrix and promotes the phosphorylation of cytoskeletal proteins such as paxillin and talin, which together play a role in the regulation of cell morphology and migration. In vascular smooth muscle cells (VSMCs), Ang II rapidly increases the phosphorylation of FAK, and losartan inhibits the phosphorylation of FAK and further Ang II-mediated VSMC migration [70]. AGTR1 also influences RhoA, whose function is to activate G_12/13_ and the subsequent interaction between its G subunit and RhoGEF, thereby further activating RhoA and leading to the stimulation of ROCK [71].

CXCR4/SDF-1α activates members of the FAK family of protein tyrosine kinases and further upregulates RhoA to stimulate ROCK, which promotes cell contractility and migration [72–75]. Therefore, we clarified the role of CXCR4 in AGTR1-mediated cell migration through the FAK/RhoA pathway by knocking down CXCR4 in AGTR1^high^ cells. For the first time, we revealed that AGTR1 overexpression increased FAK/RhoA signaling molecules, which could be decreased by siCXCR4 (Figure 8).

In conclusion, we found a new role of AGTR1-mediated tumor metastasis in our study. For the first time, we reveal that inhibition of AGTR1 by losartan reduces lymph node metastasis through CXCR4/SDF-1α. Our findings provide evidence for clinical treatments using ARBs by targeting lymph node metastasis in breast cancer patients with hypertension. Both ARBs and CXCR4 inhibitors have been translated into clinical trials in cancer patients, and additional clinical trials are needed to accelerate the development of breast cancer treatment strategies.

## METHODS

### Sample collection

Breast cancer samples were acquired from the pathology department, and the patients did not receive preoperative radiotherapy or chemotherapy. The obtained specimens and clinicopathological data (age, histological grade, tumor size, lymph node metastasis, ER, PR, HER2 status) were conducted in accordance with the ethical standards and according to the Declaration of Helsinki and according to national and international guidelines and were approved by the Ethics Committee of the Medical Faculty of Tongji Medical College. The samples were used to prepare tissue microarray slides and were then subjected to AGTR1 IHC staining Supplementary Table 1.

### Immunohistochemistry and HE staining

Sections were deparaffinized in xylene three successive times and then hydrated in different percentages of ethanol (100%, 90% and 75%). Slides were soaked in citrate buffer and boiled for 15 min at 100°C in a microwave oven. After cooling to room temperature, the slides were placed into 3% hydrogen peroxide for 20 min to block endogenous peroxide activity and then incubated with 5% BSA for 30 min. The samples were stained with primary antibodies at 4°C overnight. After treating with secondary antibody and diaminobenzidine (DAB) for 4 min in a dark room, hematoxylin was used to stain the nucleus. The slides were placed on coverslips and visualized under a light microscope.

The immunohistochemical score (IHS) was calculated by multiplying an estimate of the percentage of immunoreactive cells (quantity score) by an estimate of the staining intensity (0, negative; 1, weak; 2, moderate and 3, strong). An HSCORE >2 was considered high expression, while an HSCORE ≤2 was considered low expression.

Lymph nodes were embedded in paraffin for histopathological examination. After tissue sectioning, mounting onto slides and staining with HE, histological changes were assessed by two independent pathologists and were visualized using a light microscope.

### Cells

Human breast cancer cell lines MDA-MB-231, MCF7 and 4T1 were obtained from the Cancer Center, Union Hospital, Tongji Medical College, Huazhong University of Science and Technology (Wuhan, China) and cultured according to ATCC guidelines. MDA-MB-231 cells were cultured in L-15 media (BOSTER, China) at 37°C without CO_2_ and authenticated at XY Biotechnology Co, Ltd., in June 2016 (ABI 3730XL Genetic Analyzer, GeneAmp PCR System 9700). MCF7 cells were incubated in DMEM media (Gibco, USA) in a humidified incubator at 37°C and 5% CO_2_ and authenticated at Guangzhou Cellcook Biotech Co., Ltd., in June 2018 (PowerPlexTM16HS System). 4T1 cells were maintained in RPMI-1640 medium (Gibco, USA). However, there is currently no public reference database to match the genetic profile of mouse-origin 4T1 cells.

### RNA isolation and RT-PCR

Lymph node (ground into cell suspension) and breast cancer cell RNAs were extracted and reverse transcribed to cDNA using a reverse transcriptional kit (TaKaRa Bio, Inc.). Then, a SYBR Prime Script RT-PCR Kit (TaKaRa Bio, Inc.) was used to perform RT-PCR on the Step One Plus Real-Time PCR System. The primer sequences are shown in the following box.

**Table:** 

**Primer name**	**Primer sequence**
Human AGTR1 (f)	GATGATTGTCCCAAAGCTGG
Human AGTR1 (r)	TAGGTAATTGCCAAAGGGCC
Human CXCR4 (f)	AGCTGTTGGTGAAAAGGTGGTCTATG
Human CXCR4 (r)	GCGCTTCTGGTGGCCCTTGGAGT
Mouse SDF-1α (f)	GCCTCCAAACGCATGCTT
Mouse SDF-1α (r)	ATTGGTCCGTCAGGCTACAGA
Human GAPDH (f)	GACCACAGTCCATGACATCACT
Human GAPDH (r)	TCCACCACCCTGTTGCTGTAG
Mouse GAPDH (f)	CAGCAACTCCCACTCTTCCAC
Mouse GAPDH (r)	TGGTCCAGGGTTTCTTACTC

### Protein extraction and Western blotting

Proteins in tumor tissues (ground into cell suspension) and breast cancer cells were extracted using a mixed lysis buffer. After centrifugation at 14,000 rpm for 25 min, the supernatants were obtained to detect concentrations using a BCA kit. Protein samples (50 μg) were separated by SDS-PAGE and transferred to PVDF membranes (Millipore), which were then blocked with 5% BSA for 1 h. The membranes were incubated with primary antibodies at 4°C overnight, followed by incubation with secondary antibody for 1 h at room temperature. Proteins were visualized using an ECL detection system.

### Xenograft murine model

Balb/c nu/nu mice (3–4 weeks old) were purchased from Beijing Huafukang Bioscience Co., Inc., and Balb/c mice (6–8 weeks old) were purchased from Liaoning Changsheng Biotechnology Co., Ltd., in China and raised in the SPF animal laboratory. All animal experiments were conducted in accordance with the recommendations of the Policy on the Humane Care and Use of Laboratory Animals and approved by the Ethics Committee of HUST. Then, 50 μl of a MDA-MB-231 cell suspension (5×10^6^) with 50 μl of Matrigel and 100 μl of a 4T1 cell suspension (1×10^6^) were injected into the fourth left mammary fat pad. Tumor volume was calculated by the formula length×width^2^×0.5. The mice were randomly subdivided into five groups (8 mice/group): (1) the control group: saline 150 μl i.g.; (2) the losartan group: losartan 150 μl of 40 mg/kg/d i.g. (3) the mock group: MOCK cells+saline i.p.; (4) the AGTR1^high^ group: AGTR1^high^ cells+saline i.p.; and (5) the AGTR1^high^+AMD3100 group: AGTR1^high^ cells+AMD3100 (2.5 mg/kg/d, Sigma, St. Louis, MO, USA) i.p. After two weeks (MDA-MB-231 tumors) or one week (4T1 tumors) of tumor implantation, saline or losartan were given to the mice orally for consecutive days. The mice were sacrificed when first tumor volume reached 2000 mm^3^.

### BLI

Cells expressing firefly luciferase were seeded into 96-well plates. After adherence and the addition of D-luciferin (Xenogen, 150 mg/kg) to each well, the cells were screened by IVIS. Mice with firefly luciferase were anaesthetized and injected with D-luciferin (i.p.). After 10 min, the mice were placed in the IVIS to monitor the tumor size. To detect lymph node metastasis, proper axillary, accessory axillary, subiliac and popliteal lymph nodes of each mouse were resected for indirect BLI. The number of positive lymph nodes and firefly luciferase signals in each group were calculated.

### Cell proliferation assay

MOCK and AGTR1^high^ cells (8×10^3^ cells/well) were seeded in 96-well plates and treated with different doses of losartan (0, 100, 200, 300 μM) for 24 h, 48 h, and 72 h Supplementary Figures 5-9. CCK8 reagents were added to each well for 1 h, and the OD value was expressed as the absorbance at 450 nm.

### Transwell assay

MDA-MB-231 and MCF7 cells were seeded into transwell (Corning Costar) upper chambers with 200 μl of serum-free FBS medium and/or 100 μM losartan for migration and Matrigel invasive assays, while 600 μl of 20% FBS medium and/or 100 μM losartan was added to the lower chambers. After incubation for 16 h (MDA-MB-231) or 48 h (MCF7) at 37°C, the upper chambers were soaked in 4% paraformaldehyde and then stained with crystal violet. The cells in the upper chamber were completely removed with cotton swabs, and the cells on the reverse side of the membrane were imaged and counted under a microscope.

### siRNA transfection

Cells were seeded into 6-well plates at a density of 2 × 10^5^ cells/well and incubated overnight. A total of 5 μl of siRNA was added to 500 μl of Opti-MEM with RNAiMAX in each well according to the manufacturer's instructions. After 48 h, RNA and protein were extracted for testing the transfection efficiency. The siRNA sequences were as follows:

siAGTR1-1: GCAGTAGCCAGCAATTTGA

siAGTR1-2: ATAAGAAGGTTCAGATCCA

siCXCR4-1: CAGCUAACACAGAUGUAAATT

siCXCR4-2: GCGUGUAGUGAAUCACGUATT

### Lentivirus transfection

AGTR1 lentivirus (Huameng Biotechnology, Ltd.) was transfected into luciferase-expressing or non-luciferase-expressing MDA-MB-231 cells for animal experiments or cell experiments, respectively. After transfection, puromycin (2 μg/ml) was used to select luciferase-expressing cells and hygromycin (600 μl/ml) for AGTR1^high^ cells. The transfection efficiency was detected using PCR and Western blotting.

### Actin polymerization assay

The seeded MDA-MB-231 cells on sterile cover slips were untreated or treated with 100 μM losartan for 24 h, and SDF-1α was added for 20 min. Then, the cells were fixed in 4% formaldehyde for 20 min and incubated in 0.1% Triton X-100 with 5% BSA for 30 min. After staining with 100 nM rhodamine phalloidin, the actin filaments in the cells were captured and analyzed using a Zeiss confocal photomicroscope (Olympus FluoView FV1000).

### Reagents and antibodies

One losartan potassium tablet (50 mg, 461.01 g/mol, Merck Sharp & Dohme (Australia) Pty., Ltd.) was dissolved in 9375 μl of saline for intragastric administration, and one tablet was dissolved in 1084.6 μl of PBS for cell experiments.

The following primary antibodies were used: anti-CXCR4 (WB: ABclonal, A1303; IHC: A12534); anti-FAK (CST, 3285); anti p-FAK (CST, 3282); anti-AGTR1 (LifeSpan, LS-B4614-50); anti-RhoA (Santa Cruz, sc-418); anti-ROCK1 (Santa Cruz, sc-374388); anti-ROCK2 (ABclonal, A5698); MLC (Abcam, 3672); and p-MLC (Abcam, 3675).

### Statistical analyses

All data are presented as the mean±standard deviation (SD). Student *t* test (2-tailed, unpaired) was used for significance analysis. The *P* value<0.05 was considered significant.

## Supplementary Material

Supplementary Figures

Supplementary Table

## References

[1] Müller A, Homey B, Soto H, Ge N, Catron D, Buchanan ME, McClanahan T, Murphy E, Yuan W, Wagner SN, Barrera JL, Mohar A, Verástegui E, Zlotnik A. Involvement of chemokine receptors in breast cancer metastasis. Nature. 2001; 410:50–56. 10.1038/35065016 11242036

[2] Fisher B, Bauer M, Wickerham DL, Redmond CK, Fisher ER, Cruz AB, Foster R, Gardner B, Lerner H, Margolese R, Poisson R, Shibata H, Volk H. Relation of number of positive axillary nodes to the prognosis of patients with primary breast cancer. An NSABP update. Cancer. 1983; 52:1551–57. 10.1002/1097-0142(19831101)52:9<1551::AID-CNCR2820520902>3.0.CO;2-3 6352003

[3] Carter CL, Allen C, Henson DE. Relation of tumor size, lymph node status, and survival in 24,740 breast cancer cases. Cancer. 1989; 63:181–87. 10.1002/1097-0142(19890101)63:1<181::AID-CNCR2820630129>3.0.CO;2-H 2910416

[4] Elston CW, Ellis IO. Pathological prognostic factors in breast cancer. I. The value of histological grade in breast cancer: experience from a large study with long-term follow-up. Histopathology. 1991; 19:403–10. 10.1111/j.1365-2559.1991.tb00229.x 1757079

[5] American Joint Committee on Cancer. Manual for Staging of Cancer, 3rd edition. Editors Beahrs O, Henson D, Hutter R, Myers M. 1988 10.1097/00000421-198812000-00027

[6] Leong SP, Cady B, Jablons DM, Garcia-Aguilar J, Reintgen D, Jakub J, Pendas S, Duhaime L, Cassell R, Gardner M, Giuliano R, Archie V, Calvin D, et al. Clinical patterns of metastasis. Cancer Metastasis Rev. 2006; 25:221–32. 10.1007/s10555-006-8502-8 16770534

[7] Galimberti V, Cole BF, Zurrida S, Viale G, Luini A, Veronesi P, Baratella P, Chifu C, Sargenti M, Intra M, Gentilini O, Mastropasqua MG, et al, and Veronesi U, for the International Breast Cancer Study Group Trial 23-01 investigators. IBCSG 23-01 randomised controlled trial comparing axillary dissection versus no axillary dissection in patients with sentinel node micrometastases. Lancet Oncol. 2013; 14:297. 10.1016/S1470-2045(13)70035-4 23491275 PMC3935346

[8] Jagsi R, Chadha M, Moni J, Ballman K, Laurie F, Buchholz TA, Giuliano A, Haffty BG. Radiation field design in the ACOSOG Z0011 (Alliance) Trial. J Clin Oncol. 2014; 32:3600–06. 10.1200/JCO.2014.56.5838 25135994 PMC4220042

[9] Pereira ER, Jones D, Jung K, Padera TP. The lymph node microenvironment and its role in the progression of metastatic cancer. Semin Cell Dev Biol. 2015; 38:98–105. 10.1016/j.semcdb.2015.01.008 25620792 PMC4397158

[10] Zlotnik A, Yoshie O. Chemokines: a new classification system and their role in immunity. Immunity. 2000; 12:121–27. 10.1016/S1074-7613(00)80165-X 10714678

[11] Campbell JJ, Butcher EC. Chemokines in tissue-specific and microenvironment-specific lymphocyte homing. Curr Opin Immunol. 2000; 12:336–41. 10.1016/S0952-7915(00)00096-0 10781407

[12] Ruddle NH. Lymphatic vessels and tertiary lymphoid organs. J Clin Invest. 2014; 124:953–59. 10.1172/JCI71611 24590281 PMC3934190

[13] Kesler CT, Liao S, Munn LL, Padera TP. Lymphatic vessels in health and disease. Wiley Interdiscip Rev Syst Biol Med. 2013; 5:111–24. 10.1002/wsbm.1201 23209022 PMC3527689

[14] Luker KE, Luker GD. Functions of CXCL12 and CXCR4 in breast cancer. Cancer Lett. 2006; 238:30–41. 10.1016/j.canlet.2005.06.021 16046252

[15] Phillips RJ, Burdick MD, Lutz M, Belperio JA, Keane MP, Strieter RM. The stromal derived factor-1/CXCL12-CXC chemokine receptor 4 biological axis in non-small cell lung cancer metastases. Am J Respir Crit Care Med. 2003; 167:1676–86. 10.1164/rccm.200301-071OC 12626353

[16] Yasuoka H, Tsujimoto M, Yoshidome K, Nakahara M, Kodama R, Sanke T, Nakamura Y. Cytoplasmic CXCR4 expression in breast cancer: induction by nitric oxide and correlation with lymph node metastasis and poor prognosis. BMC Cancer. 2008; 8:340. 10.1186/1471-2407-8-340 19025611 PMC2642845

[17] Kang H, Watkins G, Douglas-Jones A, Mansel RE, Jiang WG. The elevated level of CXCR4 is correlated with nodal metastasis of human breast cancer. Breast. 2005; 14:360–67. 10.1016/j.breast.2004.12.007 16216737

[18] Liu J, Liao S, Huang Y, Samuel R, Shi T, Naxerova K, Huang P, Kamoun W, Jain RK, Fukumura D, Xu L. PDGF-D improves drug delivery and efficacy via vascular normalization, but promotes lymphatic metastasis by activating CXCR4 in breast cancer. Clin Cancer Res. 2011; 17:3638–48. 10.1158/1078-0432.CCR-10-2456 21459800 PMC3107920

[19] Liu D, Li L, Zhang XX, Wan DY, Xi BX, Hu Z, Ding WC, Zhu D, Wang XL, Wang W, Feng ZH, Wang H, Ma D, Gao QL. SIX1 promotes tumor lymphangiogenesis by coordinating TGFβ signals that increase expression of VEGF-C. Cancer Res. 2014; 74:5597–607. 10.1158/0008-5472.CAN-13-3598 25142796

[20] Wei JC, Yang J, Liu D, Wu MF, Qiao L, Wang JN, Ma QF, Zeng Z, Ye SM, Guo ES, Jiang XF, You LY, Chen Y, et al. Tumor-associated Lymphatic Endothelial Cells Promote Lymphatic Metastasis By Highly Expressing and Secreting SEMA4C. Clin Cancer Res. 2017; 23:214–24. 10.1158/1078-0432.CCR-16-0741 27401250

[21] Deshayes F, Nahmias C. Angiotensin receptors: a new role in cancer? Trends Endocrinol Metab. 2005; 16:293–99. 10.1016/j.tem.2005.07.009 16061390

[22] Arrieta O, Guevara P, Escobar E, García-Navarrete R, Pineda B, Sotelo J. Blockage of angiotensin II type I receptor decreases the synthesis of growth factors and induces apoptosis in C6 cultured cells and C6 rat glioma. Br J Cancer. 2005; 92:1247–52. 10.1038/sj.bjc.6602483 15785746 PMC2361987

[23] Fogarty DJ, Sánchez-Gómez MV, Matute C. Multiple angiotensin receptor subtypes in normal and tumor astrocytes in vitro. Glia. 2002; 39:304–13. 10.1002/glia.10117 12203396

[24] Fujimoto Y, Sasaki T, Tsuchida A, Chayama K. Angiotensin II type 1 receptor expression in human pancreatic cancer and growth inhibition by angiotensin II type 1 receptor antagonist. FEBS Lett. 2001; 495:197–200. 10.1016/S0014-5793(01)02377-8 11334891

[25] Ishimatsu S, Itakura A, Okada M, Kotani T, Iwase A, Kajiyama H, Ino K, Kikkawa F. Angiotensin II augmented migration and invasion of choriocarcinoma cells involves PI3K activation through the AT1 receptor. Placenta. 2006; 27:587–91. 10.1016/j.placenta.2005.07.001 16122787

[26] Rodrigues-Ferreira S, Abdelkarim M, Dillenburg-Pilla P, Luissint AC, di-Tommaso A, Deshayes F, Pontes CL, Molina A, Cagnard N, Letourneur F, Morel M, Reis RI, Casarini DE, et al. Angiotensin II facilitates breast cancer cell migration and metastasis. PLoS One. 2012; 7:e35667. 10.1371/journal.pone.0035667 22536420 PMC3334979

[27] Sacewicz I, Wiktorska M, Wysocki T, Niewiarowska J. Mechanisms of cancer angiogenesis. Postepy Hig Med Dosw. 2009; 63:159–68. 19502677

[28] Keibel A, Singh V, Sharma MC. Inflammation, microenvironment, and the immune system in cancer progression. Curr Pharm Des. 2009; 15:1949–55. 10.2174/138161209788453167 19519435

[29] Pinter M, Jain RK. Targeting the renin-angiotensin system to improve cancer treatment: implications for immunotherapy. Sci Transl Med. 2017; 9. 10.1126/scitranslmed.aan5616 28978752 PMC5928511

[30] Chauhan VP, Martin JD, Liu H, Lacorre DA, Jain SR, Kozin SV, Stylianopoulos T, Mousa AS, Han X, Adstamongkonkul P, Popović Z, Huang P, Bawendi MG, et al. Angiotensin inhibition enhances drug delivery and potentiates chemotherapy by decompressing tumour blood vessels. Nat Commun. 2013; 4:2516. 10.1038/ncomms3516 24084631 PMC3806395

[31] Diop-Frimpong B, Chauhan VP, Krane S, Boucher Y, Jain RK. Losartan inhibits collagen I synthesis and improves the distribution and efficacy of nanotherapeutics in tumors. Proc Natl Acad Sci USA. 2011; 108:2909–14. 10.1073/pnas.1018892108 21282607 PMC3041115

[32] De Paepe B, Verstraeten VL, De Potter CR, Vakaet LA, Bullock GR. Growth stimulatory angiotensin II type-1 receptor is upregulated in breast hyperplasia and in situ carcinoma but not in invasive carcinoma. Histochem Cell Biol. 2001; 116:247–54. 10.1007/s004180100313 11685554

[33] Singh A, Nunes JJ, Ateeq B. Role and therapeutic potential of G-protein coupled receptors in breast cancer progression and metastases. Eur J Pharmacol. 2015; 763:178–83. 10.1016/j.ejphar.2015.05.011 25981295 PMC4784721

[34] Okamoto K, Tajima H, Nakanuma S, Sakai S, Makino I, Kinoshita J, Hayashi H, Nakamura K, Oyama K, Nakagawara H, Fujita H, Takamura H, Ninomiya I, et al. Angiotensin II enhances epithelial-to-mesenchymal transition through the interaction between activated hepatic stellate cells and the stromal cell-derived factor-1/CXCR4 axis in intrahepatic cholangiocarcinoma. Int J Oncol. 2012; 41:573–82. 10.3892/ijo.2012.1499 22664794

[35] Rovito D, Gionfriddo G, Barone I, Giordano C, Grande F, De Amicis F, Lanzino M, Catalano S, Andò S, Bonofiglio D. Ligand-activated PPARγ downregulates CXCR4 gene expression through a novel identified PPAR response element and inhibits breast cancer progression. Oncotarget. 2016; 7:65109–24. 10.18632/oncotarget.11371 27556298 PMC5323141

[36] Oh E, Kim JY, Cho Y, An H, Lee N, Jo H, Ban C, Seo JH. Overexpression of angiotensin II type 1 receptor in breast cancer cells induces epithelial-mesenchymal transition and promotes tumor growth and angiogenesis. Biochim Biophys Acta. 2016; 1863:1071–81. 10.1016/j.bbamcr.2016.03.010 26975580

[37] Tomar A, Schlaepfer DD. Focal adhesion kinase: switching between GAPs and GEFs in the regulation of cell motility. Curr Opin Cell Biol. 2009; 21:676–83. 10.1016/j.ceb.2009.05.006 19525103 PMC2754589

[38] Guan JL. Integrin signaling through FAK in the regulation of mammary stem cells and breast cancer. IUBMB Life. 2010; 62:268–76. 10.1002/iub.303 20101634 PMC2848709

[39] Parri M, Chiarugi P. Rac and Rho GTPases in cancer cell motility control. Cell Commun Signal. 2010; 8:23. 10.1186/1478-811X-8-23 20822528 PMC2941746

[40] Catalano S, Campana A, Giordano C, Gyorffy B, Tarallo R, Rinaldi A, Bruno G, Ferraro A, Romeo F, Lanzino M, Naro F, Bonofiglio D, Andò S, et al. Expression and Function of Phosphodiesterase Type 5 in Human Breast Cancer Cell Lines and Tissues: Implications for Targeted Therapy. Clin Cancer Res. 2016; 22:2271–82. 10.1158/1078-0432.CCR-15-1900 26667489

[41] Vinson GP, Barker S, Puddefoot JR. The renin-angiotensin system in the breast and breast cancer. Endocr Relat Cancer. 2012; 19:R1–19. 10.1530/ERC-11-0335 22180497

[42] George AJ, Thomas WG, Hannan RD. The renin-angiotensin system and cancer: old dog, new tricks. Nat Rev Cancer. 2010; 10:745–59. 10.1038/nrc2945 20966920

[43] Sipahi I, Debanne SM, Rowland DY, Simon DI, Fang JC. Angiotensin-receptor blockade and risk of cancer: meta-analysis of randomised controlled trials. Lancet Oncol. 2010; 11:627–36. 10.1016/S1470-2045(10)70106-6 20542468 PMC4070221

[44] Bangalore S, Kumar S, Kjeldsen SE, Makani H, Grossman E, Wetterslev J, Gupta AK, Sever PS, Gluud C, Messerli FH. Antihypertensive drugs and risk of cancer: network meta-analyses and trial sequential analyses of 324,168 participants from randomised trials. Lancet Oncol. 2011; 12:65–82. 10.1016/S1470-2045(10)70260-6 21123111

[45] Suganuma T, Ino K, Shibata K, Kajiyama H, Nagasaka T, Mizutani S, Kikkawa F. Functional expression of the angiotensin II type 1 receptor in human ovarian carcinoma cells and its blockade therapy resulting in suppression of tumor invasion, angiogenesis, and peritoneal dissemination. Clin Cancer Res. 2005; 11:2686–94. 10.1158/1078-0432.CCR-04-1946 15814650

[46] Kinoshita J, Fushida S, Harada S, Yagi Y, Fujita H, Kinami S, Ninomiya I, Fujimura T, Kayahara M, Yashiro M, Hirakawa K, Ohta T. Local angiotensin II-generation in human gastric cancer: correlation with tumor progression through the activation of ERK1/2, NF-kappaB and survivin. Int J Oncol. 2009; 34:1573–82. 10.3892/ijo_00000287 19424575

[47] Röcken C, Lendeckel U, Dierkes J, Westphal S, Carl-McGrath S, Peters B, Krüger S, Malfertheiner P, Roessner A, Ebert MP. The number of lymph node metastases in gastric cancer correlates with the angiotensin I-converting enzyme gene insertion/deletion polymorphism. Clin Cancer Res. 2005; 11:2526–30. 10.1158/1078-0432.CCR-04-1922 15814629

[48] Röcken C, Röhl FW, Diebler E, Lendeckel U, Pross M, Carl-McGrath S, Ebert MP. The angiotensin II/angiotensin II receptor system correlates with nodal spread in intestinal type gastric cancer. Cancer Epidemiol Biomarkers Prev. 2007; 16:1206–12. 10.1158/1055-9965.EPI-05-0934 17548686

[49] Puddefoot JR, Udeozo UK, Barker S, Vinson GP. The role of angiotensin II in the regulation of breast cancer cell adhesion and invasion. Endocr Relat Cancer. 2006; 13:895–903. 10.1677/erc.1.01136 16954438

[50] Ekambaram P, Lee JL, Hubel NE, Hu D, Yerneni S, Campbell PG, Pollock N, Klei LR, Concel VJ, Delekta PC, Chinnaiyan AM, Tomlins SA, Rhodes DR, et al. The CARMA3-Bcl10-MALT1 Signalosome Drives NFκB Activation and Promotes Aggressiveness in Angiotensin II Receptor-Positive Breast Cancer. Cancer Res. 2018; 8:1225–40. 10.1158/0008-5472.CAN-17-1089 29259013 PMC6436094

[51] Hirakawa S, Brown LF, Kodama S, Paavonen K, Alitalo K, Detmar M. VEGF-C-induced lymphangiogenesis in sentinel lymph nodes promotes tumor metastasis to distant sites. Blood. 2007; 109:1010–17. 10.1182/blood-2006-05-021758 17032920 PMC1785149

[52] Hirakawa S, Kodama S, Kunstfeld R, Kajiya K, Brown LF, Detmar M. VEGF-A induces tumor and sentinel lymph node lymphangiogenesis and promotes lymphatic metastasis. J Exp Med. 2005; 201:1089–99. 10.1084/jem.20041896 15809353 PMC2213132

[53] Wirzenius M, Tammela T, Uutela M, He Y, Odorisio T, Zambruno G, Nagy JA, Dvorak HF, Ylä-Herttuala S, Shibuya M, Alitalo K. Distinct vascular endothelial growth factor signals for lymphatic vessel enlargement and sprouting. J Exp Med. 2007; 204:1431–40. 10.1084/jem.20062642 17535974 PMC2118625

[54] Gu Y, Qi X, Guo S. Lymphangiogenesis induced by VEGF-C and VEGF-D promotes metastasis and a poor outcome in breast carcinoma: a retrospective study of 61 cases. Clin Exp Metastasis. 2008; 25:717–25. 10.1007/s10585-008-9180-4 18512120

[55] Proulx ST, Luciani P, Derzsi S, Rinderknecht M, Mumprecht V, Leroux JC, Detmar M. Quantitative imaging of lymphatic function with liposomal indocyanine green. Cancer Res. 2010; 70:7053–62. 10.1158/0008-5472.CAN-10-0271 20823159 PMC3398157

[56] Paget S. The distribution of secondary growths in cancer of the breast. 1889. Cancer Metastasis Rev. 1989; 8:98–101. 2673568

[57] Qian CN, Berghuis B, Tsarfaty G, Bruch M, Kort EJ, Ditlev J, Tsarfaty I, Hudson E, Jackson DG, Petillo D, Chen J, Resau JH, Teh BT. Preparing the “soil”: the primary tumor induces vasculature reorganization in the sentinel lymph node before the arrival of metastatic cancer cells. Cancer Res. 2006; 66:10365–76. 10.1158/0008-5472.CAN-06-2977 17062557

[58] Ben-Baruch A. Site-specific metastasis formation: chemokines as regulators of tumor cell adhesion, motility and invasion. Cell Adh Migr. 2009; 3:328–33. 10.4161/cam.3.4.9211 19550136 PMC2802740

[59] Grande F, Barone I, Aiello F, Brancale A, Cancellieri M, Badolato M, Chemi F, Giordano C, Vircillo V, Bonofiglio D, Garofalo A, Andò S, Catalano S. Identification of novel 2-(1H-indol-1-yl)-benzohydrazides CXCR4 ligands impairing breast cancer growth and motility. Future Med Chem. 2016; 8:93–106. 10.4155/fmc.15.176 26807787

[60] Wani N, Nasser MW, Ahirwar DK, Zhao H, Miao Z, Shilo K, Ganju RK. C-X-C motif chemokine 12/C-X-C chemokine receptor type 7 signaling regulates breast cancer growth and metastasis by modulating the tumor microenvironment. Breast Cancer Res. 2014; 16:R54. 10.1186/bcr3665 24886617 PMC4076630

[61] Houshmand P, Zlotnik A. Therapeutic applications in the chemokine superfamily. Curr Opin Chem Biol. 2003; 7:457–60. 10.1016/S1367-5931(03)00086-3 12941419

[62] Thiery JP, Acloque H, Huang RY, Nieto MA. Epithelial-mesenchymal transitions in development and disease. Cell. 2009; 139:871–90. 10.1016/j.cell.2009.11.007 19945376

[63] Yang J, Mani SA, Donaher JL, Ramaswamy S, Itzykson RA, Come C, Savagner P, Gitelman I, Richardson A, Weinberg RA. Twist, a master regulator of morphogenesis, plays an essential role in tumor metastasis. Cell. 2004; 117:927–39. 10.1016/j.cell.2004.06.006 15210113

[64] Rizzo P, Novelli R, Benigni A, Remuzzi G. Inhibiting angiotensin-converting enzyme promotes renal repair by modulating progenitor cell activation. Pharmacol Res. 2016; 108:16–22. 10.1016/j.phrs.2016.04.009 27095084

[65] Xu C, Zhao H, Chen H, Yao Q. CXCR4 in breast cancer: oncogenic role and therapeutic targeting. Drug Des Devel Ther. 2015; 9:4953–64. 10.2147/DDDT.S84932 26356032 PMC4560524

[66] Pernas S, Martin M, Kaufman PA, Gil-Martin M, Gomez Pardo P, Lopez-Tarruella S, Manso L, Ciruelos E, Perez-Fidalgo JA, Hernando C, Ademuyiwa FO, Weilbaecher K, Mayer I, et al. Balixafortide plus eribulin in HER2-negative metastatic breast cancer: a phase 1, single-arm, dose-escalation trial. Lancet Oncol. 2018; 19:812–24. 10.1016/S1470-2045(18)30147-5 29706375

[67] Vicente-Manzanares M, Vitón M, Sánchez-Madrid F. Measurement of the levels of polymerized actin (F-actin) in chemokine-stimulated lymphocytes and GFP-coupled cDNA transfected lymphoid cells by flow cytometry. Methods Mol Biol. 2004; 239:53–68. 10.1385/1-59259-435-2:53 14573909

[68] Martin SK, Diamond P, Williams SA, To LB, Peet DJ, Fujii N, Gronthos S, Harris AL, Zannettino AC. Hypoxia-inducible factor-2 is a novel regulator of aberrant CXCL12 expression in multiple myeloma plasma cells. Haematologica. 2010; 95:776–84. 10.3324/haematol.2009.015628 20015878 PMC2864384

[69] Samarendra H, Jones K, Petrinic T, Silva MA, Reddy S, Soonawalla Z, Gordon-Weeks A. A meta-analysis of CXCL12 expression for cancer prognosis. Br J Cancer. 2017; 117:124–35. 10.1038/bjc.2017.134 28535157 PMC5520200

[70] Dong X, Yu LG, Sun R, Cheng YN, Cao H, Yang KM, Dong YN, Wu Y, Guo XL. Inhibition of PTEN expression and activity by angiotensin II induces proliferation and migration of vascular smooth muscle cells. J Cell Biochem. 2013; 114:174–82. 10.1002/jcb.24315 22887358

[71] Ohtsu H, Suzuki H, Nakashima H, Dhobale S, Frank GD, Motley ED, Eguchi S. Angiotensin II signal transduction through small GTP-binding proteins: mechanism and significance in vascular smooth muscle cells. Hypertension. 2006; 48:534–40. 10.1161/01.HYP.0000237975.90870.eb 16923993

[72] Prasad A, Fernandis AZ, Rao Y, Ganju RK. Slit protein-mediated inhibition of CXCR4-induced chemotactic and chemoinvasive signaling pathways in breast cancer cells. J Biol Chem. 2004; 279:9115–24. 10.1074/jbc.M308083200 14645233

[73] Fernandis AZ, Prasad A, Band H, Klösel R, Ganju RK. Regulation of CXCR4-mediated chemotaxis and chemoinvasion of breast cancer cells. Oncogene. 2004; 23:157–67. 10.1038/sj.onc.1206910 14712221

[74] Lee BC, Lee TH, Avraham S, Avraham HK. Involvement of the chemokine receptor CXCR4 and its ligand stromal cell-derived factor 1alpha in breast cancer cell migration through human brain microvascular endothelial cells. Mol Cancer Res. 2004; 2:327–38. 10.1158/0008-5472.can-04-3309 15235108

[75] Guo J, Yu X, Gu J, Lin Z, Zhao G, Xu F, Lu C, Ge D. Regulation of CXCR4/AKT-signaling-induced cell invasion and tumor metastasis by RhoA, Rac-1, and Cdc42 in human esophageal cancer. Tumour Biol. 2016; 37:6371–78. 10.1007/s13277-015-4504-x 26631033

