# Supplementary material for: AGTR1 promotes lymph node metastasis in breast cancer by upregulating CXCR4/SDF-1α and inducing cell migration and invasion
Source: Aging (Albany NY). 2019 Jun 19;11(12):3969–92. doi: 10.18632/aging.102032 (PMC6628987; doi:10.18632/aging.102032)
Supplement: Supplementary Figures [file aging-11-102032-s002.pdf]

SUPPLEMENTARY FIGURES

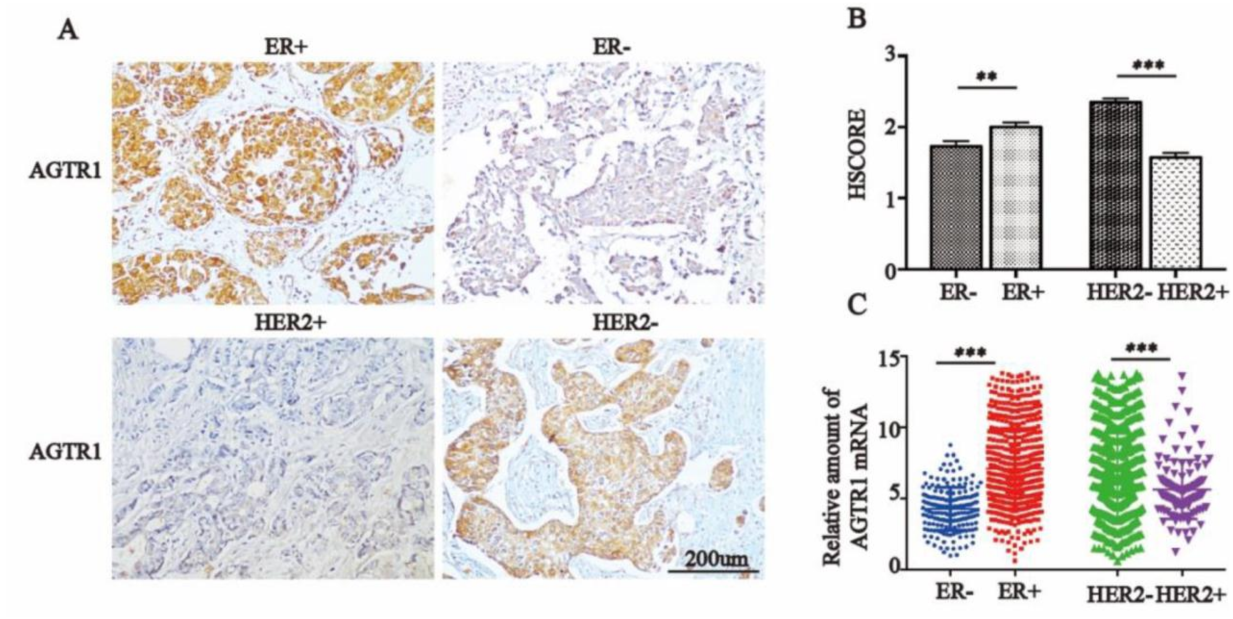

Supplementary Figure 1. AGTR1 is highly expressed in ER+ or HER2- tumour tissues. (**A**) The tumour samples with different ER and HER2- statuses were subjected to immunohistochemical staining with anti-AGTR1. (**B**) HSCORE of AGTR1 protein expression in breast cancer tissues with different ER and HER2 statuses. **, P<0.01; ***P<0.001. (**C**) Relative amount of AGTR1 mRNA in cancer tissues with different ER and HER2 statuses in the TCGA database. ***, P<0.001.

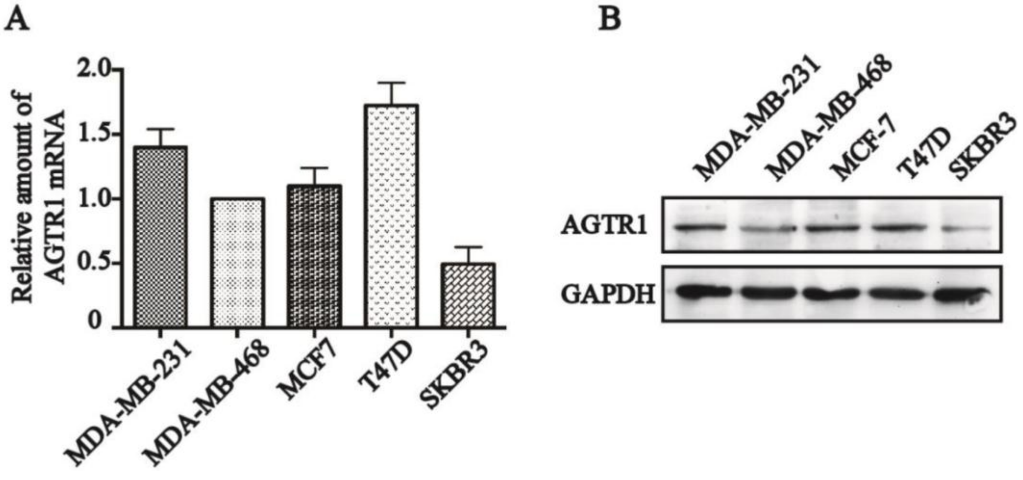

Supplementary Figure 2. AGTR1 expression in breast cancer cells. (**A**) AGTR1 mRNA and (**B**) protein expression in MDA-MB-231, MDA-MB-468, MCF-7, T47D and SKBR3 breast cancer cells.

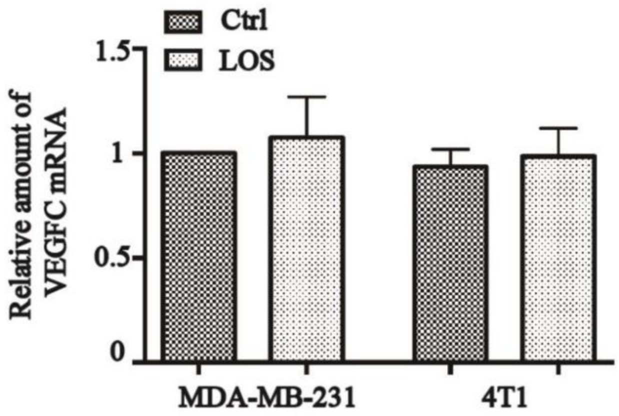

Supplementary Figure 3. Level of VEGF-C mRNA in MDA-MB-231 and 4T1 mouse tumours.

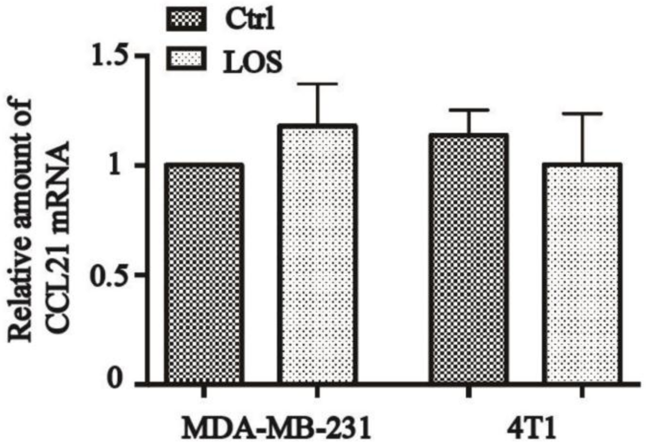

Supplementary Figure 4. Level of CCL21 mRNA in MDA-MB-231 and 4T1 mouse lymph nodes.

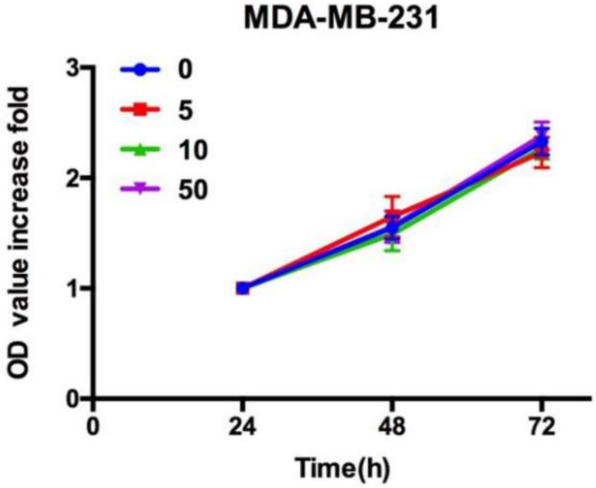

Supplementary Figure 5. MDA-MB-231 cell viability after treatment with different concentrations of LOS (0, 5, 10, 50 μM) by CCK8.

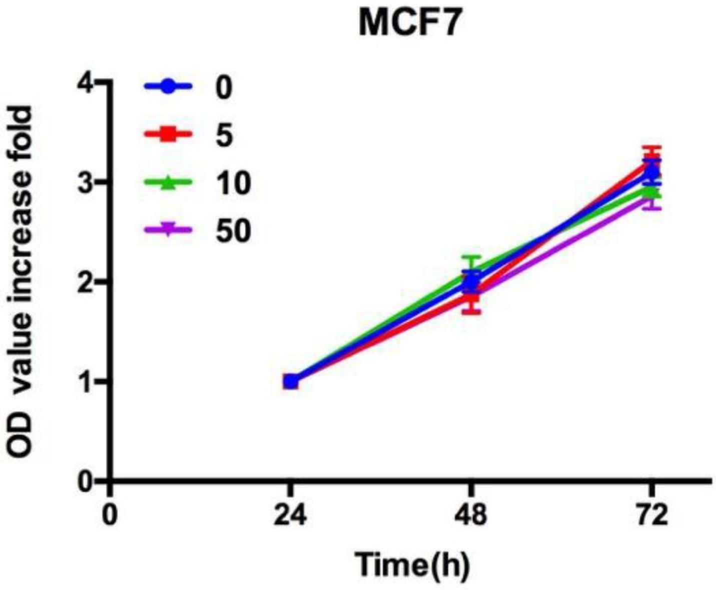

Supplementary Figure 6. MCF7 cell viability after treatment with different concentrations of LOS (0, 5, 10, 50 μM) by CCK8.

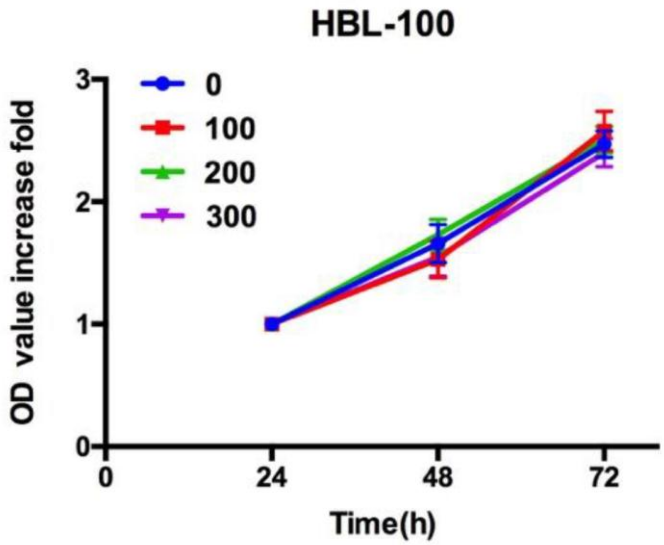

Supplementary Figure 7. HBL-100 cell viability after treatment with different concentrations of LOS (0, 100, 200, 300 μM) by CCK8.

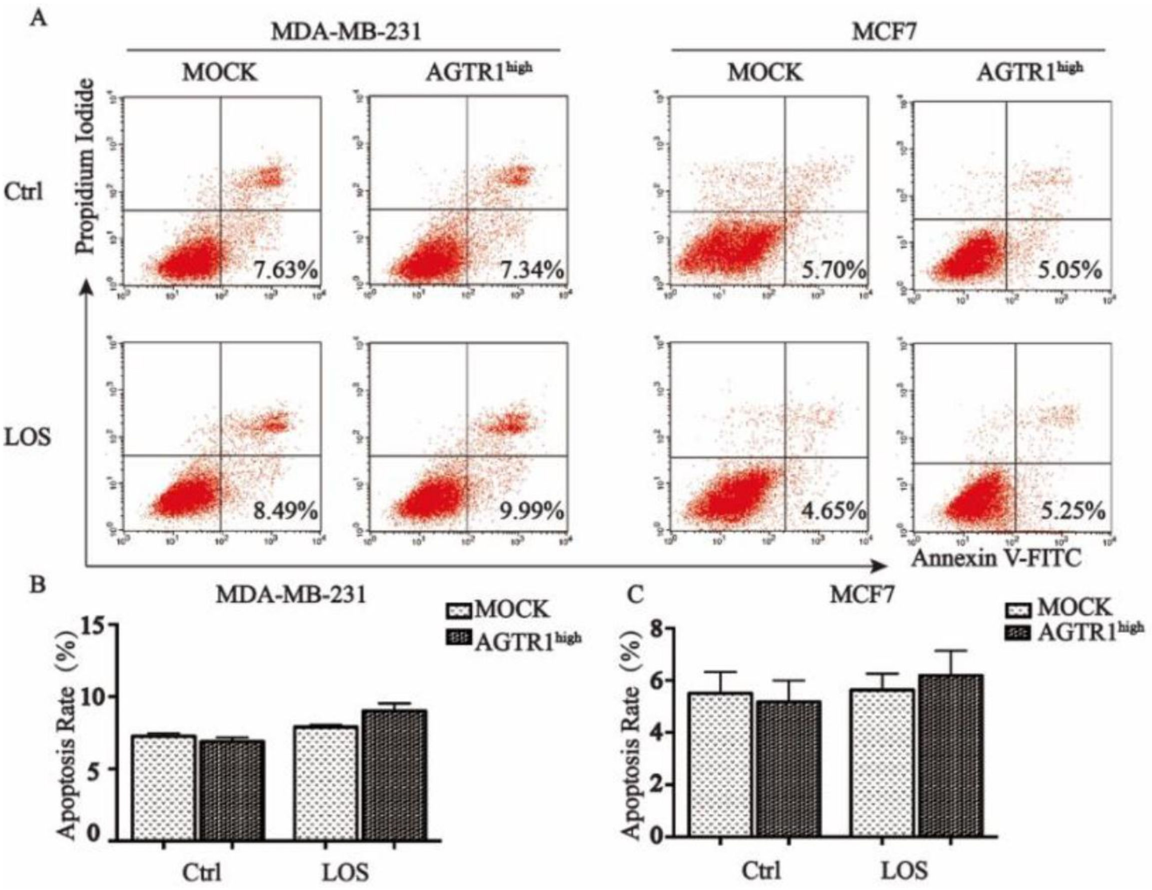

Supplementary Figure 8. MOCK and AGTR1high cell apoptosis with LOS 100 μM.

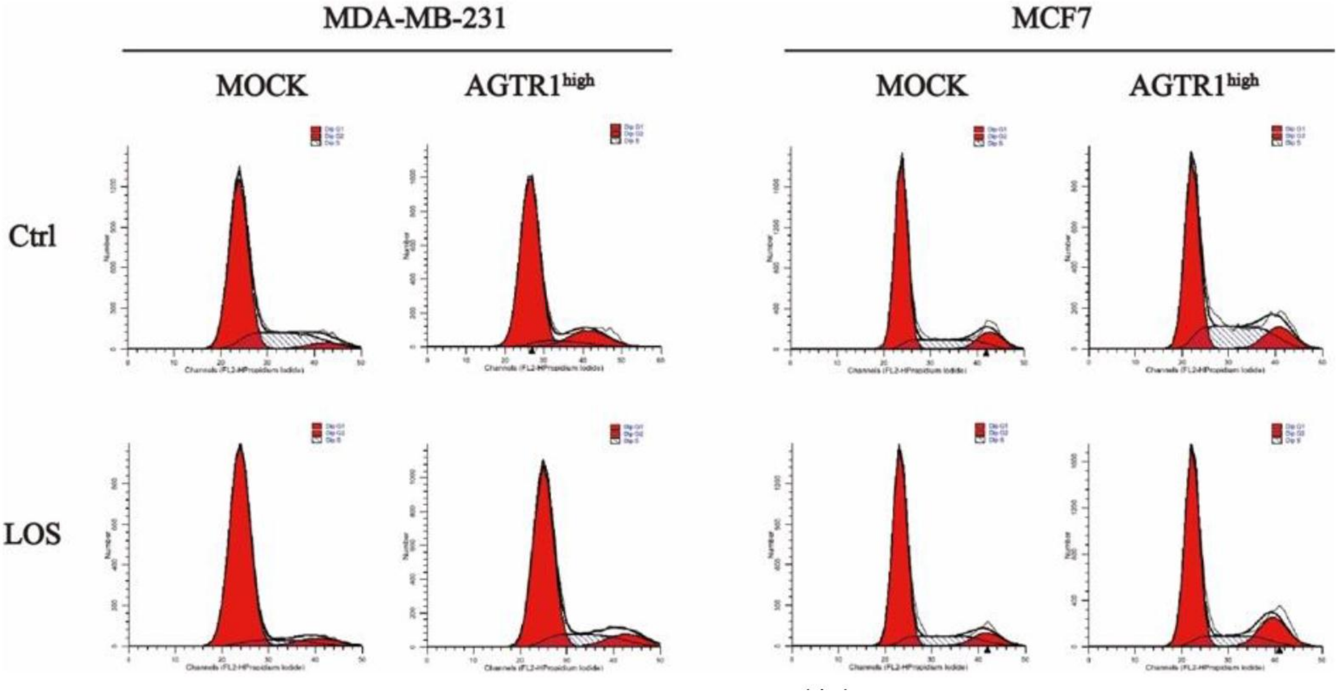

Supplementary Figure 9. MOCK and AGTR1high cell cycle with LOS 100 μM.
