# Supplementary material for: AGTR1 promotes lymph node metastasis in breast cancer by upregulating CXCR4/SDF-1α and inducing cell migration and invasion
Source: Aging (Albany NY). 2019 Jun 19;11(12):3969–92. doi: 10.18632/aging.102032 (PMC6628987; doi:10.18632/aging.102032)
Supplement: Supplementary Table [file aging-11-102032-s001.pdf]

SUPPLEMENTARY TABLE

Supplementary Table 1. Statistical table of the tissue samples and oncomine database for the lymph node-positive and lymph node-negative groups.

	LN−			LN+		
	**N**	**Mean**	**SD**	**N**	**Mean**	**SD**
**IHC**	148	1.52	1.03	127	2.16	0.56
**Oncomine**	17	−0.88	0.21	607	0.12	0.55
